# Editorial: Viral Hepatitis: Pathophysiology, Prevention, and Control

**DOI:** 10.3389/fcimb.2021.633580

**Published:** 2021-08-26

**Authors:** Cheng Kao, Milan Surjit, C. T. Ranjith-Kumar

**Affiliations:** ^1^Molecular and Cellular Biochemistry, Indiana University, Bloomington, IN, United States; ^2^Vaccine and Infectious Disease Research Center, Translational Health Science and Technology Institute (THSTI), Faridabad, India; ^3^University School of Biotechnology, Guru Gobind Singh Indraprastha University, New Delhi, India

**Keywords:** viral hepatitis, HAV, HBV, HCV, HDV, HEV

Viral hepatitis, characterized by liver inflammation and damage, is among the leading human global health threats (World Health Organization, 2012). Billions of people worldwide have been infected with hepatitis viruses. Millions worldwide are living with viral hepatitis and over 1.4 million deaths occur annually as a result of liver cirrhosis and cancer (World Health Organization, 2016). Notably, many infected individuals are unaware of their infection and can transmit the virus to others.

The majority of viral hepatitis, alphabetically referred to as Types A, B, C, D, and E, are caused by five different viruses. In addition, human adenoviruses are also known to cause hepatitis in immunocompromised individuals. These viruses are from distinct taxonomic grouping, have different infectivity, replication strategies, reservoirs, and pathogenesis. In turn, the course of the disease, epidemiology, prevention and treatment could differ.

The enveloped hepatitis B virus (HBV) and hepatitis C virus (HCV) cause both acute and chronic liver disease. Together HBV and HCV account for ~ 90% of the fatalities. HCV, a member of the *Flaviviridae* family of positive-strand RNA virus, infects through exposures to virus-containing blood or body fluids that contain blood. HBV is a member of the *Hepadnaviridae* family with a reverse-transcribed partially double-stranded genome. It infects through puncture of the skin or mucosal contact with infectious blood or body fluids. Hepatitis D virus (HDV) contains a circular single-stranded negative-sense RNA genome and it co-infects people along with hepatitis B or infect people who are already chronically infected by HBV. The envelope of HDV contains the HBV envelope proteins and it uses the same cellular receptor for entry as HBV (Yan et al., 2012). HDV and HBV co-infection can speed up liver disease progression.

Hepatitis A virus (HAV) and hepatitis E virus (HEV) from feces are nonenveloped viruses. Interestingly, HEV from blood contain membrane from exosomes and are classified as quasi-enveloped (Nagashima et al., 2017). Both viruses spread through close contact with an infected individual or through ingestion of virus-contaminated food or drink. HAV and HEV belong to the family of *Picronaviridae* and *Hepeviridae*, respectively, and both possess single-stranded positive-sense RNA genomes (Aggarwal, 2013; Lemon et al., 2017). It is important to note that HEV also infects a number of animals that serve as reservoirs and ingestion of undercooked meats can lead to transmission (Doceul et al., 2016). HAV and HEV typically lead to self-limiting diseases, although the illness could last for a few months. In a small percentage of people, especially immunocompromised individuals, specific genotypes of HEV can cause chronic infection (Hoofnagle et al., 2012). Severe acute infections can also lead to a small percentage of fatalities, and fatalities could be higher in pregnant women (Khuroo and Kamili, 2003).

Significant progress to prevent infection by hepatitis viruses has been made. Highly effective and safe prophylactic vaccines are available for hepatitis A and B. For hepatitis B, vaccination of newborns weighing over 2 kg is recommended (Schillie et al., 2018). Proper vaccination will also produce essentially life-long immunity (Loader et al., 2019). Preventing HBV infection will also protect against HDV infection. For chronically infected hepatitis B patients, the vaccine will not cure the infection, but family members and associates who receive the vaccine can be protected from infection. A recombinant vaccine against HEV is available in China (Zhu et al., 2010). A vaccine is not available for hepatitis C. Prevention of infection should focus on improved education, hygiene, improving infrastructure, and healthcare practices.

It is important to have therapies for viral hepatitis. Thus far, therapy development have primarily focused on hepatitis B and C. HBV can now be controlled with direct-acting antivirals (DAAs), although the virus cannot be effectively eliminated. Additional therapies with multiple modalities that can achieve functional cures, the sustained loss of the HBV surface antigen, are actively pursued by the biotech and pharma industry (Lok et al., 2017; Tang et al., 2017). Treatment for hepatitis C has shifted from the use of immunomodulators and the general viral inhibitor ribavirin to molecules that target the viral replication proteins (Scheel and Rice, 2013). Several combinations of DAAs can effectively elimination pan-genotypic HCV with very low rate of viral relapse (Zoratti et al). The general recommendation for individuals infected with HAV is to rest and avoid stressors that can exacerbate symptoms. As discussed above, there is more urgent need to treat HEV infection. Currently, recombinant interferon and ribavirin are available, but effective DAAs remain to be identified (Netzler et al).

This Research Topic contains both reviews and original articles that deal with a range of topics on viral hepatitis. Despite the significant advances made in Hepatitis B research, HBV continues to be a serious threat to public health worldwide. In their review, Steinmann et al. put hepatitis B into a historical context and the lessons on proper formulation of vaccines. The authors emphasized on the high heat tolerance of HBV and suggested the importance of proper washing and disinfection to minimize HBV contamination and overall safety. The entry of HBV into host cells is not completely understood and is an area that requires more attention. Though sodium-taurocholate cotransporting polypeptide (NTCP) was shown to be a functional receptor for HBV, other molecules are reported to aid in HBV infection. To better understand HBV infectivity, Hu et al. documented that E-cadherin, an adhesion molecule that is abundant in epithelial tissue, can interact with the HBV receptor to affect its localization in cells and thus impact HBV entry. Interestingly, E-cadherin binds only to the glycosylated form of NTCP, which is important for it to act as the HBV receptor, and thus preferentially regulates the membrane localization of glycosylated NTCP.

Three articles dealt with viral pathogenesis. Liu et al. used transcriptome analysis to demonstrate that the HCV Core protein expressed from a chimeric virus can induce interleukin-32 expression. Use of specific inhibitors in their study pointed to the involvement of PI3K/AKT pathway in IL32 induction. Authors suggested that the HCV core protein dependent IL32 expression leads to the elevated inflammation associated with severe hepatitis. One of the peculiar disease manifestations of HEV is its involvement in neurological disorders. Not much is known about how HEV induces neurological complications. Tian et al. showed that HEV could cause mitochondrial-associated apoptosis in cultured human brain cells and in the brains of HEV-infected gerbils. This further induced the production of high amounts of pro-inflammatory cytokines possibly explaining the observed extrahepatic injuries associated with HEV infection. In the review by Sehgal et al., the link between gut microbiota and the severity of viral hepatitis was examined. The authors focused on the role of bacterial pathogen-associated molecular patterns in activating innate immune receptors resulting in the NFκB signaling in hepatitis B and C dependent hepatitis. Fecal microbiota transplant could be an alternative method of treatment and management for viral hepatitis.

Four articles deal with therapeutic development and possible therapeutic molecules. Khan et al. reviewed the diverse HCV replicons that were instrumental to the development of HCV drugs and threw light on drug resistance. The replicons for all the major HCV genotypes, including subgenomic and full-length replicons, and their importance in HCV drug discovery are discussed. For HEV, a promising drug target is the protease. HEV encoded protease was suggested to be a papain-like cysteine protease as well as chymotrypsin like protease. In their work, Saraswat et al. showed that the HEV protease was able to process the ORF1 polyprotein. Furthermore, the authors used biochemical assays and in silico modeling to show that the HEV protease is indeed a papain-like cysteine protease. Also in this Research Topic, the modulation of polyamine synthesis as a possible antiviral strategy was investigated. Polyamines are known to play a role in the replication of many RNA and DNA viruses. Mao et al. showed that polyamines help in HBV replication and an inhibitor of polyamine synthesis can inhibit HBV by increasing the proteasome-mediated degradation of the Core protein. Finally, in this Research Topic, Wang et al. focused on another virus whose infection can cause hepatitis, the human adenovirus. They developed a mouse monoclonal antibody against one of the major capsid proteins of human adenovirus (HAdV-7) and showed that this antibody has a potent neutralizing activity.

The original articles and reviews presented in this Research Topic showcase the work of diverse group of researchers from different parts of the world on viral hepatitis. The knowledge provided will contribute to the development of interference strategies and treatment for viral hepatitis.

C. Cheng Kao

Milan Surjit

C. T. Ranjith-Kumar

## Author Contributions

CK, MS and CR-K were the topic editors of this Research Topic and co-wrote the editorial. All the authors contributed to the article and approved the submitted version.

## Conflict of Interest

The authors declare that the research was conducted in the absence of any commercial or financial relationships that could be construed as a potential conflict of interest.

## Publisher’s Note

All claims expressed in this article are solely those of the authors and do not necessarily represent those of their affiliated organizations, or those of the publisher, the editors and the reviewers. Any product that may be evaluated in this article, or claim that may be made by its manufacturer, is not guaranteed or endorsed by the publisher.
